# Hemimegalencephaly With Diffuse Cortical Malformation in a Child With Drug-Resistant Infantile Epileptic Spasms: A Case Report

**DOI:** 10.7759/cureus.115613

**Published:** 2026-09-01

**Authors:** Boutaina Essaher, Yahya Sqalli Houssaini, Samia Amar, Hassan En Nouali, Meriem Boui

**Affiliations:** 1 Radiology, Mohammed V Military Teaching Hospital, Rabat, MAR; 2 Radiology, Mohammed V University, Rabat, MAR

**Keywords:** hemimegalencephaly, hemispherotomy, infantile epileptic spasms, malformation of cortical development, west syndrome

## Abstract

Hemimegalencephaly is a rare congenital malformation of cortical development involving hamartomatous overgrowth of part or all of one cerebral hemisphere. It commonly presents during infancy with early-onset, drug-resistant seizures, developmental impairment, and contralateral motor deficits. Infantile epileptic spasms may be an initial manifestation. Brain magnetic resonance imaging (MRI) is central to diagnosis and surgical planning.

We report the case of an 11-month-old boy with West syndrome and severe developmental delay whose epileptic spasms persisted despite treatment with vigabatrin, sodium valproate, and levetiracetam. Brain MRI demonstrated marked asymmetric enlargement of the left cerebral hemisphere, diffuse cortical thickening, blurring of the gray-white matter differentiation, enlargement and morphological distortion of the left lateral ventricle, shallow sulcation, and extensive pachygyria with areas approaching an agyric appearance. The right cerebral hemisphere was structurally preserved. These findings were consistent with left hemimegalencephaly associated with an extensive malformation of cortical development. An anterior corpus callosotomy was performed as a palliative disconnective procedure; however, the epileptic spasms persisted, and the patient was subsequently referred for evaluation of definitive left hemispherotomy.

This case illustrates the characteristic MRI appearance of hemimegalencephaly with diffuse cortical malformation and highlights the limitations of anterior corpus callosotomy when the epileptogenic substrate involves an entire malformed hemisphere. It also emphasizes the role of imaging in guiding escalation to definitive hemispheric disconnection when seizures remain drug-resistant.

## Introduction

Hemimegalencephaly is a rare congenital malformation of cortical development characterized by hamartomatous overgrowth involving part or all of one cerebral hemisphere [1]. It results from abnormal neuronal and glial proliferation, migration, and cortical organization and is typically associated with diffuse cortical dysplasia and abnormal white matter development [2,3]. Hemimegalencephaly may occur as an isolated condition or as part of an overgrowth or neurocutaneous syndrome, including epidermal nevus syndrome, hypomelanosis of Ito, Klippel-Trenaunay syndrome, and tuberous sclerosis complex [1,2].

The clinical presentation is typically dominated by early-onset seizures, developmental delay or regression, and contralateral motor impairment [1]. Infantile epileptic spasms syndrome, historically referred to as West syndrome when the classic electroclinical triad is present, may be an initial manifestation and is characterized by epileptic spasms, hypsarrhythmia on electroencephalography (EEG), and developmental stagnation or regression [4]. Epilepsy associated with hemimegalencephaly is frequently drug-resistant, making early recognition of a unilateral structural epileptogenic substrate particularly important [5].

Magnetic resonance imaging (MRI) plays a central role in both diagnosis and therapeutic planning. Characteristic findings include hemispheric enlargement, cortical thickening, abnormal sulcation and gyration, impaired gray-white matter differentiation, ventricular distortion, and associated white matter abnormalities. MRI also allows detailed assessment of the contralateral hemisphere and infratentorial structures, which may influence surgical candidacy and prognosis [1,2,6].

In patients with severe unilateral hemispheric involvement, early surgical treatment may provide substantial seizure control and limit the detrimental effects of persistent epileptic activity on the developing contralateral hemisphere [5,7]. Hemispherotomy or functional hemispherectomy represents the definitive surgical strategy [5]. Although anatomical hemispherectomy provides extensive removal and isolation of the affected hemisphere, it is associated with greater operative morbidity. Modern hemispherotomy techniques therefore aim to achieve comparable functional disconnection while minimizing tissue resection. The principal surgical targets include interruption of the corticothalamic pathways at the internal capsule and corona radiata, disconnection or resection of the medial temporal structures, complete corpus callosotomy, and interruption of the orbitofrontal-hypothalamic pathways [8]. Successful hemispheric disconnection may reduce the adverse effects of continuous epileptic activity on the structurally preserved hemisphere and can be associated with cognitive and functional improvement [8].

Corpus callosotomy, in contrast, is a palliative disconnective procedure that reduces interhemispheric propagation and bilateral synchronization of epileptic activity without isolating the epileptogenic hemisphere itself. It may be particularly useful when disabling generalized seizures or rapid bilateral synchronization predominate, or when the epileptogenic focus cannot be reliably lateralized [9]. A partial anterior callosotomy may be selected to reduce the risk of disconnection-related morbidity; however, preserved posterior callosal fibers maintain residual interhemispheric connectivity and may permit continued seizure propagation [9].

We report this case to illustrate the characteristic MRI features of hemimegalencephaly and the persistence of epileptic spasms after anterior corpus callosotomy, highlighting the importance of complete hemispheric disconnection and the rationale for definitive hemispherotomy in severe unilateral disease.

## Case presentation

An 11-month-old boy presented to our institution in January 2026 with West syndrome, characterized by recurrent infantile epileptic spasms and severe developmental delay. Seizures had begun during the neonatal period, initially as subtle focal motor events that were retrospectively recognized as epileptic, before evolving into typical clusters of epileptic spasms occurring several times per day. Initial EEG demonstrated a disorganized high-amplitude background with multifocal spikes and sharp waves consistent with hypsarrhythmia. Subsequent recordings showed persistent multifocal epileptiform abnormalities with left hemispheric predominance and frequent bilateral synchronous discharges. Despite sequential and combined treatment with vigabatrin, sodium valproate, and levetiracetam, the spasms persisted at a frequency of approximately five to eight clusters per day.

At presentation, neurological examination revealed marked global developmental delay, axial hypotonia, and mild right-sided spastic hemiparesis, with brisker deep tendon reflexes on the right and a right extensor plantar response. The child was able to sit independently but could not walk without assistance. Fine motor skills were limited, particularly in the right hand, and no meaningful words had been acquired. No cutaneous, vascular, or somatic overgrowth features suggestive of a syndromic form of hemimegalencephaly were identified.

Brain MRI was performed on a 1.5-T system using axial fluid-attenuated inversion recovery, coronal T2-weighted, coronal inversion-recovery T1-weighted, sagittal T1-weighted, diffusion-weighted, and susceptibility-sensitive sequences, without gadolinium administration. MRI demonstrated marked asymmetric enlargement of the left cerebral hemisphere, diffuse cortical thickening with blurring of the gray-white matter differentiation, enlargement and morphological distortion of the left lateral ventricle, shallow sulcation, and extensive pachygyria with some regions approaching an agyric appearance (Figures 1-3).

**Figure 1:**
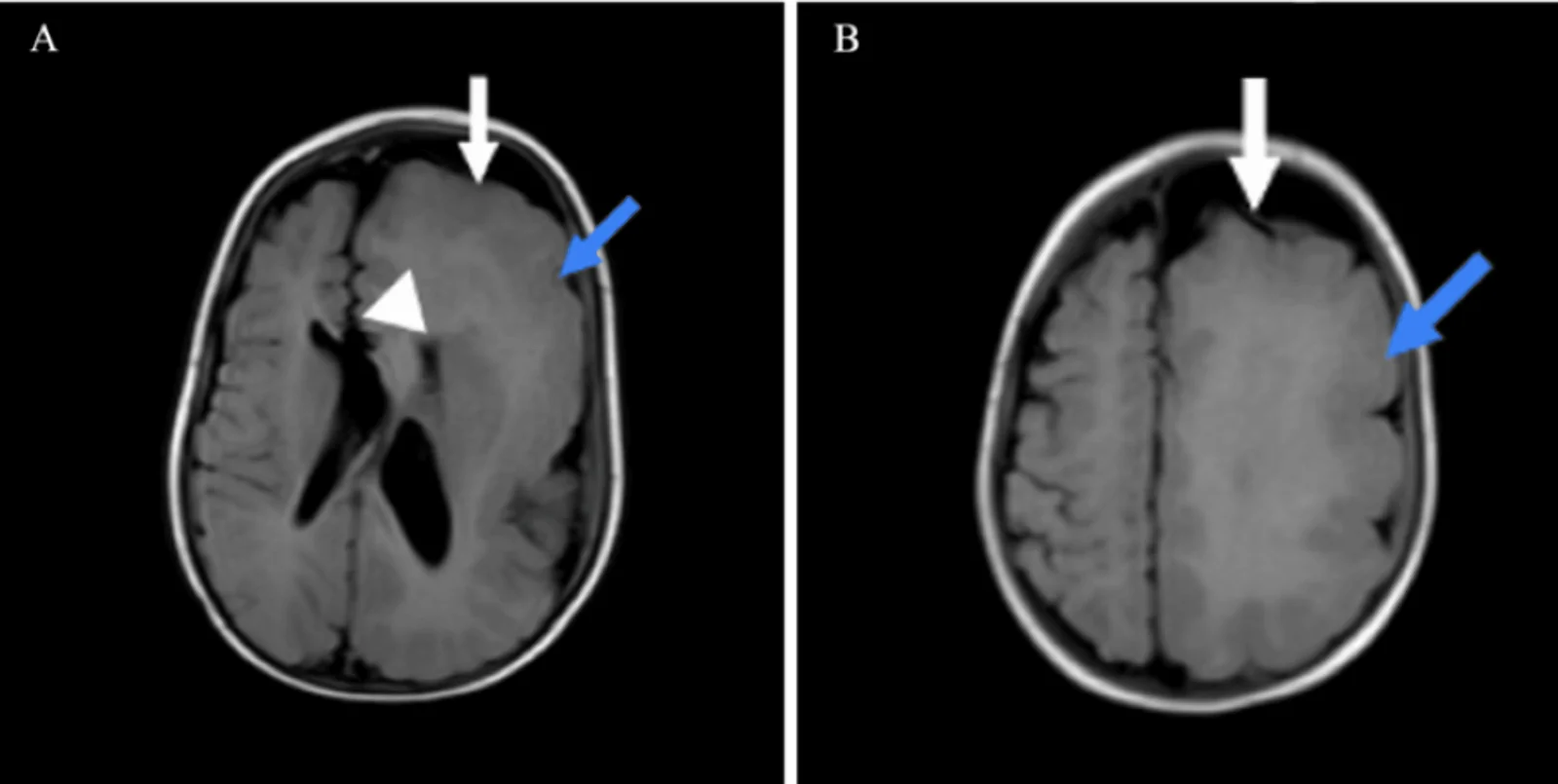
Axial FLAIR images obtained at the level of the lateral ventricles (A) and centrum semiovale (B) demonstrate marked enlargement of the left cerebral hemisphere (white arrows), diffuse cortical thickening (blue arrows), and blurring of the gray-white matter junction. The left lateral ventricle is enlarged and dysmorphic (white arrowhead). The right cerebral hemisphere is comparatively preserved. FLAIR: fluid-attenuated inversion recovery

**Figure 2:**
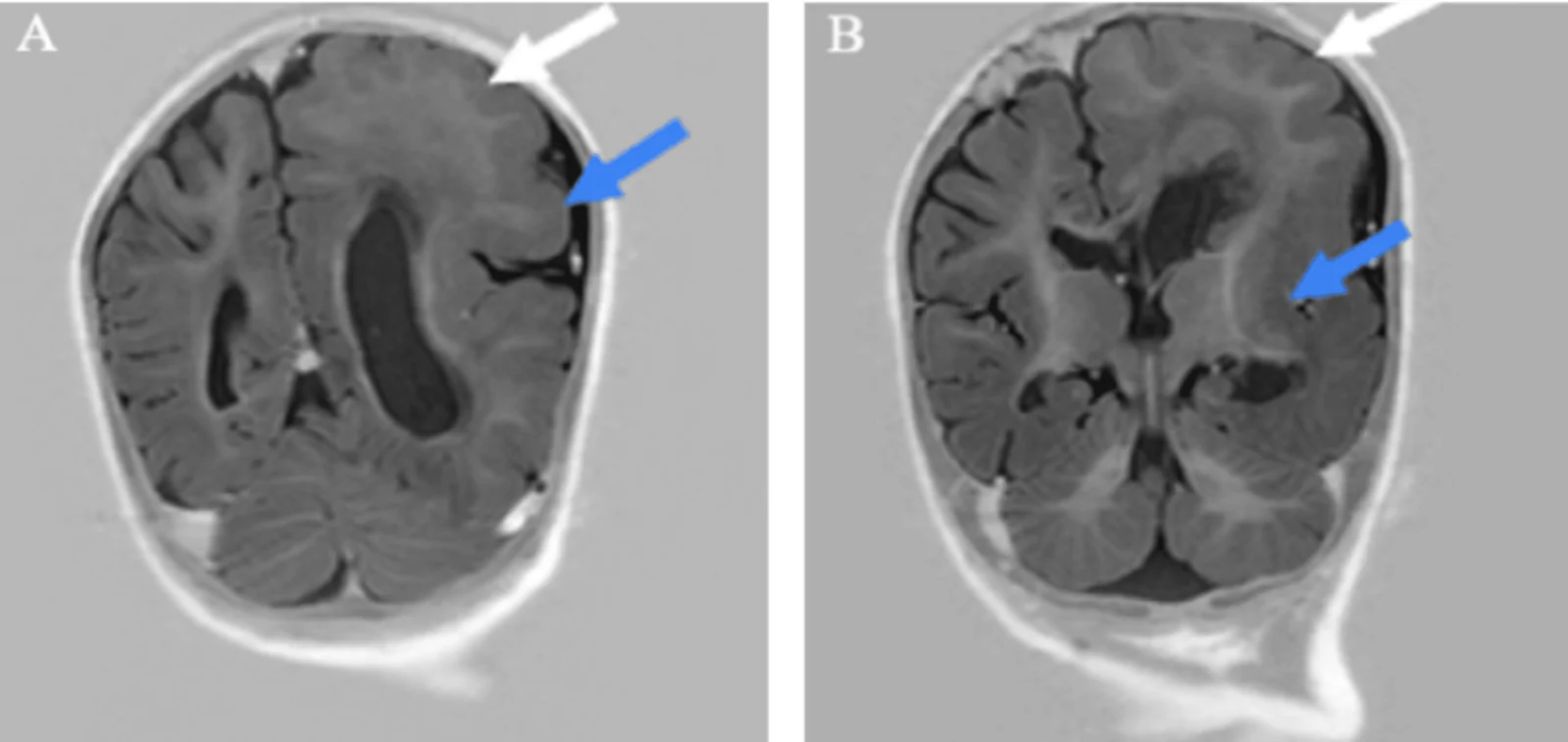
Coronal inversion-recovery T1-weighted images at two levels (A, B) show enlargement of the left cerebral hemisphere with broad dysplastic gyri, shallow sulci (white arrows), and diffuse cortical thickening (blue arrows). The right cerebral hemisphere demonstrates preserved architecture.

**Figure 3:**
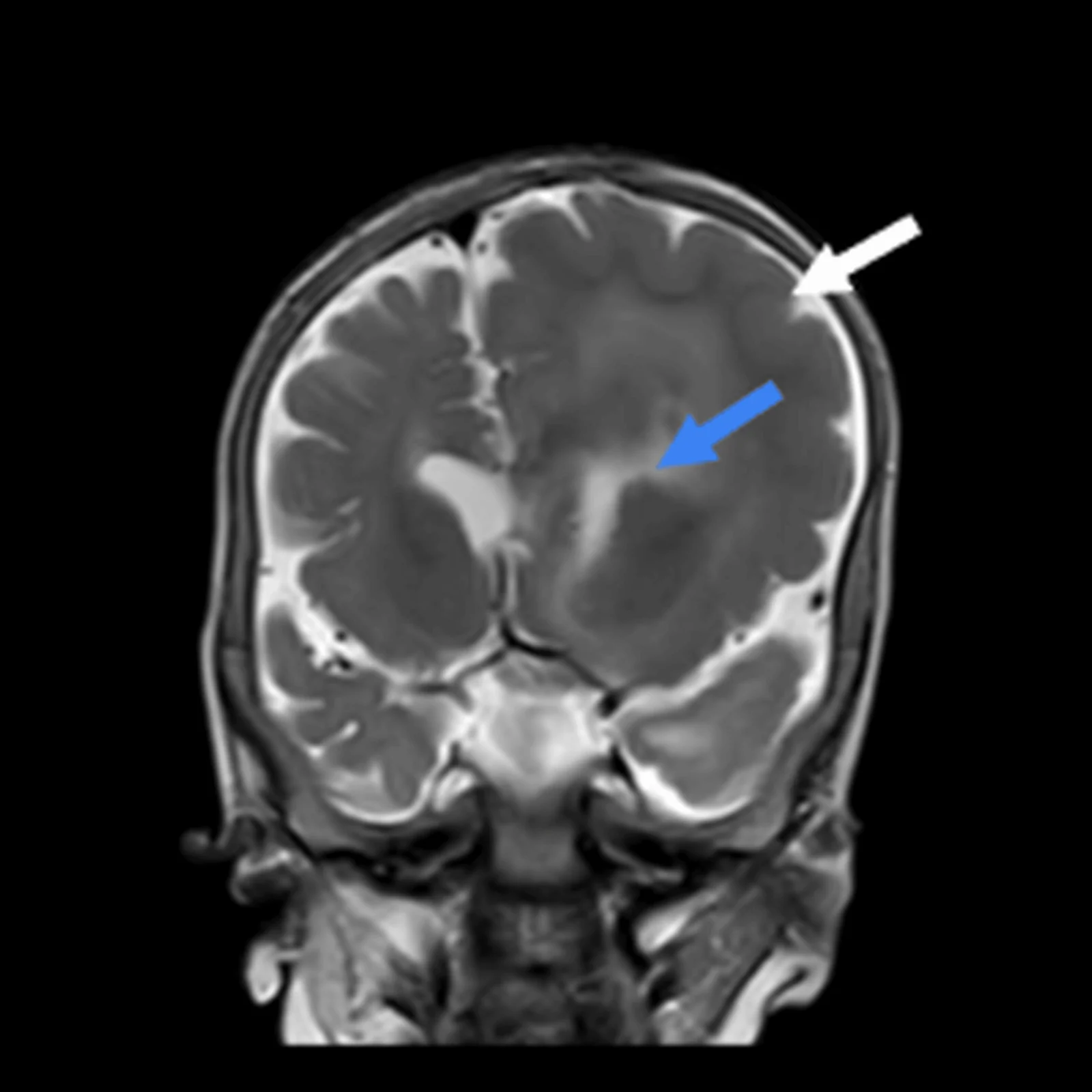
Coronal T2-weighted image showing diffuse enlargement of the left cerebral hemisphere (white arrow) associated with enlargement and irregular configuration of the left lateral ventricle (blue arrow).

The right cerebral hemisphere showed preserved volume, gyral morphology, and gray-white matter differentiation, with no brainstem or cerebellar enlargement. Diffusion-weighted and susceptibility-sensitive sequences showed no additional abnormalities. The combination of unilateral hemispheric overgrowth, diffuse cortical malformation, abnormal gyration and sulcation, gray-white matter blurring, and ipsilateral ventricular dysmorphism was consistent with left hemimegalencephaly associated with an extensive malformation of cortical development. In the absence of recognized extracerebral manifestations or infratentorial involvement, the clinicoradiologic phenotype favored an isolated form.

Given the persistence of disabling epileptic spasms despite multidrug antiseizure therapy and the frequent bilateral synchronization of epileptic discharges on EEG, the case was discussed in a multidisciplinary epilepsy-surgery setting. An anterior corpus callosotomy with anterior commissurotomy was performed through a bifrontal craniotomy and an interhemispheric approach as a first-stage palliative disconnective procedure aimed at reducing interhemispheric propagation of epileptic activity. The immediate postoperative course was uneventful. Postoperative sagittal T1-weighted MRI confirmed interruption of the anterior corpus callosum involving the rostrum and genu, with preservation of the body and splenium (Figure 4).

**Figure 4:**
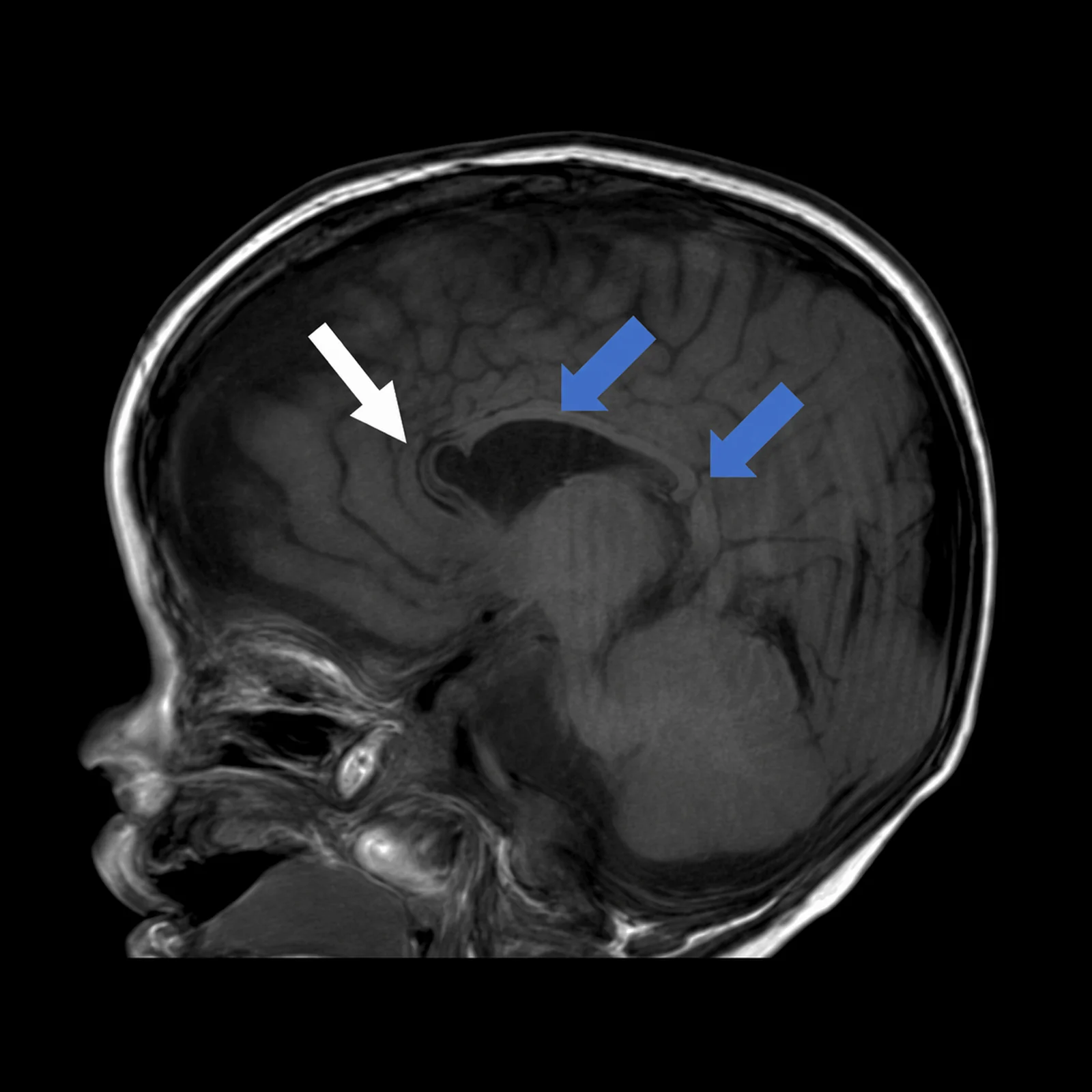
Postoperative sagittal T1-weighted image demonstrating interruption of the anterior corpus callosum, involving the rostrum and genu, with preservation of the body and splenium (blue arrows), consistent with anterior corpus callosotomy (white arrow).

Despite surgery, epileptic spasms persisted clinically, with no meaningful reduction in cluster frequency during seven months of follow-up. The preserved callosal body and splenium could have allowed residual interhemispheric propagation of epileptic activity; however, persistence of the diffusely malformed left hemispheric epileptogenic substrate remained the principal explanation for the continued seizures. Because the seizure burden remained unchanged, extension to a complete callosotomy was not favored. Instead, definitive hemispheric disconnection by hemispherotomy or functional hemispherectomy was recommended. As this procedure was not available at our institution, the patient was referred abroad for surgical management.

## Discussion

Hemimegalencephaly represents the severe end of the spectrum of developmental brain abnormalities related to dysregulated cellular growth and cortical organization. Somatic mosaic variants affecting the phosphoinositide 3-kinase-AKT-mechanistic target of rapamycin (PI3K-AKT-mTOR) signaling pathway have been identified in affected brain tissue, including variants in PIK3CA and AKT3 [10] and, subsequently, in MTOR [11], providing a molecular basis for the coexistence of hemispheric overgrowth and cortical dysplasia [3]. Three clinicoradiologic forms are commonly recognized: isolated hemimegalencephaly, syndromic hemimegalencephaly, and total hemimegalencephaly, in which enlargement also involves the ipsilateral brainstem and cerebellum [1,2]. In the present case, the absence of extracranial manifestations and infratentorial enlargement favored an isolated phenotype, although definitive molecular classification would require genetic testing.

The radiologic diagnosis relies on a constellation of imaging findings rather than hemispheric enlargement alone. Characteristic MRI features include enlargement of the affected cerebral hemisphere, enlargement and distortion of the ipsilateral lateral ventricle, enlarged gyri, loss of gray-white matter differentiation, and cortical dysplasia [1,2]. Additional findings may include thickening of the ipsilateral calvarium, contralateral displacement of the posterior falx, white matter gliosis and calcification, and accelerated myelination along the affected fiber tracts [6]. Less frequently, abnormalities may extend across the corpus callosum into the contralateral hemisphere and may be associated with developmental venous anomalies, cerebrovascular dilatation, ipsilateral olfactory nerve enlargement, ipsilateral cerebellar enlargement, and abnormal cerebellar foliation [2]. The cortex of the affected hemisphere may occasionally appear relatively preserved but more commonly demonstrates abnormalities of cortical development, including polymicrogyria, lissencephaly, agyria, pachygyria, and gray matter heterotopia [1,2].

The principal imaging differential diagnoses include extensive focal cortical dysplasia, unilateral megalencephaly in the context of syndromic overgrowth disorders, and, less commonly, mass-forming or vascular lesions. Sturge-Weber syndrome typically results in progressive unilateral cerebral atrophy rather than true hemispheric overgrowth and is usually associated with leptomeningeal angiomatosis, enlarged transmedullary veins, and cortical calcifications [1]. Dyke-Davidoff-Masson syndrome likewise results in cerebral hemiatrophy with compensatory calvarial remodeling [2]. A neoplastic process may enlarge part of a cerebral hemisphere but would not usually reproduce the diffuse developmental abnormalities of gyration, cortical architecture, and ventricular morphology observed in hemimegalencephaly. Clinical evaluation for cutaneous or segmental overgrowth features therefore remains essential because imaging alone cannot reliably exclude all syndromic associations.

Epilepsy associated with hemimegalencephaly frequently begins during the neonatal period or within the first weeks of life, reflecting the severity and diffuse nature of the underlying cortical dysplasia. This early onset, as observed in the present case, is generally earlier than in infantile epileptic spasms syndrome of other etiologies and is frequently resistant to medical treatment [4]. Persistent epileptic activity may adversely affect the development and function of the structurally preserved hemisphere through bilateral propagation and epileptic encephalopathy [7]. In patients with a severely malformed and predominantly unilateral hemisphere causing drug-resistant seizures, functional hemispherectomy and hemispherotomy are generally regarded as definitive surgical options [5,8]. Disconnective procedures are intended to isolate the epileptogenic hemisphere while reducing the long-term morbidity historically associated with anatomical hemispherectomy [8]. Published series have shown substantial rates of seizure reduction or seizure freedom, although neurodevelopmental outcomes remain variable and depend on factors such as age at seizure onset, duration of uncontrolled epilepsy, contralateral structural or functional abnormalities, and completeness of surgical disconnection [5,7].

Corpus callosotomy is a palliative procedure designed to reduce bilateral synchronization and interhemispheric propagation of epileptic discharges and may be beneficial in selected patients with epileptic spasms, particularly when more extensive surgery is not immediately feasible [9]. In the present case, MRI clearly lateralized the structural abnormality to the left hemisphere, whereas EEG showed left hemispheric predominance with frequent bilateral synchronous discharges. This mixed electroclinical pattern contributed to the initial choice of a palliative callosotomy. Unlike hemispherotomy, however, callosotomy does not functionally isolate the epileptogenic hemisphere, and persistent or asymmetric spasms after callosotomy have been proposed as an indicator of an underlying unilateral epileptogenic focus warranting further resective evaluation [12].

In the present patient, epileptic spasms persisted after anterior corpus callosotomy. Continued seizures are best explained by persistence of the diffusely malformed and intrinsically epileptogenic left hemisphere. Residual callosal connectivity through the preserved body and splenium may additionally have permitted ongoing interhemispheric propagation of epileptic activity. Together, these findings supported consideration of definitive hemispheric disconnection.

This case is instructive because it illustrates the characteristic imaging appearance of hemimegalencephaly with an extensive malformation of cortical development and highlights the therapeutic limitations of anterior callosotomy in the setting of a diffuse unilateral hemispheric malformation. An incomplete callosal section may permit residual interhemispheric propagation through preserved fibers [9,12]. More importantly, even a complete callosotomy would not eliminate the intrinsically epileptogenic malformed hemisphere itself. This distinction explains why hemispherotomy, rather than callosotomy alone, represents the definitive therapeutic option in diffuse unilateral malformations such as hemimegalencephaly [5,7,8].

A limitation of this report is the absence of a rigorous quantitative comparison between pre- and postoperative seizure frequency, as well as the lack of postoperative follow-up after hemispherotomy, because this procedure was not performed at our institution and the patient was referred elsewhere for definitive surgical management.

## Conclusions

Hemimegalencephaly should be considered in infants presenting with epileptic spasms, developmental impairment, and unilateral hemispheric enlargement. MRI is essential for confirming the diagnosis, characterizing the extent of cortical and hemispheric involvement, and assessing the contralateral hemisphere and associated structures.

Persistent epileptic spasms after anterior corpus callosotomy, without a meaningful reduction in frequency, primarily indicate ongoing epileptogenic activity within the malformed hemisphere; preserved posterior callosal fibers may additionally permit residual interhemispheric propagation. In this setting, the diffuse, drug-resistant, and predominantly unilateral nature of the epileptogenic substrate supports referral for definitive hemispheric disconnection when feasible.
